# Supplementary material for: Lateralization (handedness) in Magellanic penguins
Source: PeerJ. 2019 May 20;7:e6936. doi: 10.7717/peerj.6936 (PMC6532617; doi:10.7717/peerj.6936)
Supplement: Supplemental Information 2 — Counts from Punta Tombo, Argentina, 2014-2015. Columns: Right = blood only on the right side of the face; Left = blood only on the left side of the face; Both = blood on both sides of the face. Rows: Little = blood covering less than 1/3 of the face; Moderate = blood on more than 1/3 and less than 2/3 of the face; A lot = blood covering more than 2/3 of the face. [file peerj-07-6936-s002.docx]

|  | Right | Left | Both | Total |
| --- | --- | --- | --- | --- |
| Little | 50 | 36 | 15 | 101 |
| Moderate | 46 | 14 | 16 | 76 |
| A lot | 24 | 5 | 9 | 38 |
| Total | 120 | 55 | 40 | 215 |
